# β-Cyclodextrin Functionalized Au@Ag Core-Shell Nanoparticles: Plasmonic Sensors for Cysteamine and Efficient Nanocatalysts for Nitrobenzene-to-Aniline Conversion

**DOI:** 10.3390/bios14110544

**Published:** 2024-11-09

**Authors:** Ramar Rajamanikandan, Kandasamy Sasikumar, Heongkyu Ju

**Affiliations:** 1 Department of Physics, Gachon University, Seongnam-si 13120, Republic of Korea; chemistrmkd@gachon.ac.kr (R.R.); sasiphy2022@gachon.ac.kr (K.S.); 2 Gachon Bionano Research Institute, Gachon University, Seongnam-si 13120, Republic of Korea

**Keywords:** core-shell nanoparticles, catalytic activity, sensing ability, nitrobenzene, cysteamine, colorimetry

## Abstract

We reported the gold/silver core-shell nanoparticles (Au_core_@Ag_shell_ NPs) functionalized with β-cyclodextrin (β-CD) as versatile nano-agents demonstrated for human urine-based biosensing of cysteamine and catalytic conversion from nitrobenzene (NB) to aniline. First, the hybrid bimetallic nanoparticles, i.e., β-CD-Au_core_@Ag_shell_ NPs, constituted a colorimetric sensing platform based on localized surface plasmons, enabling cysteamine (Cyst) to be detected in a remarkably rapid manner, i.e., within 2 min, which was greatly shortened in comparison with that of our previous report. This was due largely to use of β-CD being effectively replaceable by Cyst. The detection of Cyst was demonstrated using human urine specimens in the linear range of 25–750 nM with a limit of detection of 1.83 nM. Excellent specificity in detecting Cyst was also demonstrated against potential interfering molecules. Meanwhile, the β-CD-Au_core_@Ag_shell_ NPs were demonstrated as nanocatalysts for converting NB to aniline with efficiency enhanced by more than three-fold over the pure gold nanoparticles previously reported, due to the dual functions of the structural core-shell. The demonstrated versatile features of the hybrid nanoparticles can find applications in human urine-based biosensors for Cyst detection, and in the screening of Cyst-containing drugs, while detoxicating NB for ecological protection in aqueous media.

## 1. Introduction

Noble metal nanostructures such as gold (Au) and silver (Ag) nanoparticles (NPs) have been garnering continued interest due to their unique physicochemical properties, such as the excitability of localized surface plasmon resonance (LSPR) at visible wavelengths, efficiency in electron donation for catalytic reaction, and a tremendously large ratio of surface-to-volume. The negative value of the relative permittivity of such nanostructures and their much smaller dimension compared to the wavelengths of incident light permit the resonant oscillation of electric dipoles due to surface conduction electron density oscillating coherently with electric fields of light, which is a phenomenon called LSPR [1,2,3,4,5]. Strong absorption and elastic scattering of incident light can occur as their spectral dependence is highly sensitive to the size and aspect ratio of nanoparticles [6,7,8]. Plasmon-induced enhancement of local fields around metal nanostructures enormously increases their interaction with adsorbed local analytes. This can lay the foundation for colorimetric sensing of analytes as they are adsorbed to metal nanostructures [9,10,11,12] or cause aggregation of NPs [13,14,15]. Since plasmon-based sensing platforms can be categorized into optical transducers that respond to the ambient refractive index change very sensitively, similar to optical waveguide-based refractive index sensors [16,17,18], they can benefit from immunity to external electromagnetic noise and disturbance and be further extended for the label-free detection of analytes [17,19].

Additionally, the bimetallic nanoparticles (BMNPs) that are composed of two distinct metallic elements exhibit significantly better properties (such as distinct optical, electronic, and catalytic properties) than monometallic nanoparticles. This is due to extended degrees of tunability of plasmonic and physicochemical properties [20,21,22]. In particular, BMNPs offer an extra degree of flexibility for adjusting the localized surface plasmon resonance (LSPR) wavelength by varying the composition ratio of two metals, making them more intriguing than pure MNPs [23,24]. For instance, Au (core)-Ag (shell), i.e., Au@Ag bimetallic nanoparticles are considerably stable while the local field enhancement is comparable to or even higher than Au or Ag nanoparticles [25]. The fact that Au was more chemically stable than Ag while Ag provided a higher quality factor of plasmons than gold led to the combination of both metals into a core-shell structure [26,27]. Several reports have previously established that bimetallic Au–Ag nanoparticles exhibited better performance for surface-enhanced Raman spectroscopy (SERS) and catalytic activity as compared with pure Au or Ag nanoparticles [26,27,28,29].

Cysteamine (Cyst), i.e., 2-mercaptoethylamine (HSCH_2_CH_2_NH_2_), has a low molecular weight with an aminothiol structure endogenously degraded from Coenzyme A. It played a pivotal role relevant to physiological regulation in mammals (Scheme S1) [30,31] while being mostly employed for neuroprotective drug [32,33] management of neurodegenerative conditions such as Parkinson’s and Huntington’s disorders [34]. Increased levels may result in many side effects, such as elevated alkaline phosphatase, Ehlers-Danlos syndrome, gastrointestinal bleeding or ulcers, and idiopathic intracranial hypertension (IIH), which can cause nausea, dizziness, and ringing in the ears in addition to visual loss [31,32,33]. Cyst sensing in non-laboratory settings, including home monitoring or point-of-care settings, can be extremely important for guaranteeing prompt medication treatment. Monitoring cysteamine levels outside of conventional laboratory settings can improve treatment compliance, facilitate clinical decision-making in real time, and ultimately aid in the improved management of illnesses that call for cysteamine therapy [33]. Hence, given the adverse effects associated with Cyst in clinical settings, the development of a cost-effective and user-friendly bioanalytical approach for cysteamine detection in test samples is crucial.

Cyst is currently detected using many traditional techniques. To detect Cyst in clinical settings, for example, gas chromatography [35], electrochemical [36,37], high-performance liquid chromatography [38,39], and other approaches are employed. Unfortunately, chromatographic techniques require costly chemicals and equipment and are either laborious or difficult. Optical sensing methods that are more straightforward and cost-effective strategies for Cyst sensing have been sought without compromising sensitivity and specificity. Recently, noble NPs were reported as colorimetric nanoplatforms for Cyst sensing, such as polyvinylpyrrolidone-conjugated silver nanoparticles (PVP-AgNPs) in human serum specimens [13]. However, in most cases, it took a reaction of no less than five minutes to complete for sensing, manifesting a relatively inefficient sensing reaction rate.

**Scheme 1:**
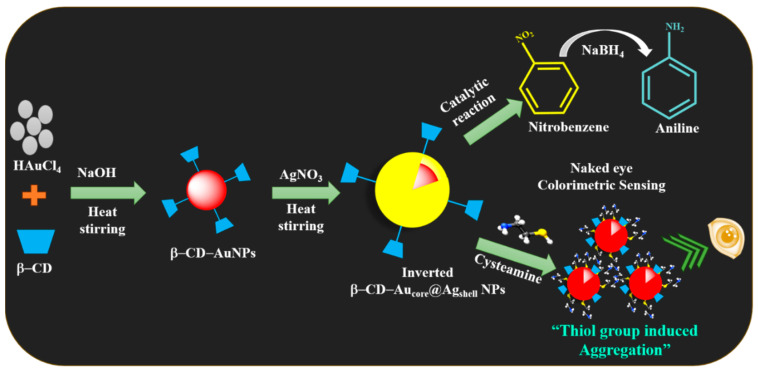
Schematic representation of this study.

In this study, we presented the core (Au)-shell (Ag) nanoparticles (Au_core_@Ag_shell_ NPs) which were functionalized with β-cyclodextrin (β-CD), as versatile nano-agents for human urine-based biosensing of cysteamine and for catalytic conversion from nitrobenzene (NB) to aniline (Scheme 1). First, we used these hybrid nanoparticles, i.e., β-CD-Au_core_@Ag_shell_ NPs, which constituted a colorimetric sensing platform based on LSPR for detecting cysteamine (Cyst) present in human urine specimens. The quantitative detection of Cyst was demonstrated in the linear range of 25–750.0 nM with a limit of detection (LOD) of 1.83 nM. We found that excellent specificity in Cyst detection was possible against other interfering molecules, including mercapto amino acids. The excellent Cyst responsivity of β-CD-Au_core_@Ag_shell_ NPs arose from the fact that the metal–thiol bonding driven by electrostatic interaction between thiol-containing Cyst and metallic nanoprobes, replaced the weakly adsorbed β-CD. This eventually aggregated NPs, altering plasmonic resonance for colorimetric sensing. This effective replacement of β-CD by Cyst enabled a reaction time as short as 2 min, which was greatly shortened in comparison with previous reports [13,40,41,42,43].

β-CD-Au_core_@Ag_shell_ NPs were also demonstrated as nanocatalysts for nitrobenzene (NB)-to-aniline conversion. It was identified that the core-shell structure produced effective catalytic conversion with its reaction constant enhanced by more than three-fold over that of pure Au NPs [44]. Consequently, the excellent ability to reduce NB while producing a somewhat useful product (aniline) for the chemical industry could lend itself to environmentally friendly nanocatalysts for removing hazardous substances such as nitroaromatics, thus manifesting the versatile proficiency of β-CD-Au_core_@Ag_shell_ NPs.

## 2. Methods and Materials

### 2.1. Chemical Reagents and Instrumentation

β-CD, silver nitrate (AgNO_3_), Cyst, tetrachloro auric acid (HAuCl_4_·3H_2_O), Sodium hydroxide (NaOH), and sodium borohydride (NaBH_4_) were purchased from Sigma Aldrich, St. Louis, MO, USA. All the chemicals were at least of analytical reagent grade and used as received. All the experiments were executed using ultra-milli-Q water. Sensing and catalytic studies were performed on a JASCO V-630 (JASCO, Tokyo, Japan) absorption spectrophotometer. The morphology of the as-prepared Au_core_@Ag_shell_ NPs (without β-CD) was identified by high-resolution electron microscopy (HR-TEM, JEOL JEM 2100, JEOL., Ltd., Tokyo, Japan). A Horiba Scientific nanoparticle analyzer SZ-100V2 (Kyoto, Japan) instrument was used to measure the average hydrodynamic diameter (dynamic light scattering) and surface charge (zeta potential) of Au_core_@Ag_shell_ NPs in the solution medium throughout an average of 32 runs. Fourier transform infrared (FT-IR) spectral data were taken from an infrared spectrometer (FTIR-4600, JASCO, Japan) via the ATR technique. X-ray photoelectron spectroscopy (XPS) was utilized to examine Au_core_@Ag_shell_ NPs with an apparatus (Multilab-2000, Thermo Scientific, New York, NY, USA) equipped with a monochromatic AlKα X-ray source (1486.6 eV) for excitation.

### 2.2. Synthesis of β-CD-Au_core_@Ag_shell_ NPs

Using the previously described procedure [45], β-cyclodextrin (β-CD)-stabilized Au_core_@Ag_shell_ NPs were developed. A 48.6 mL aqueous solution of β-CD (7.05 mM) was blended with 0.2 mL of HAuCl_4_ (10 mM). To get the pH of the mixture down to 10–12, 0.5 mL of 1.0 M NaOH was introduced after 5 min. Subsequently, this mixture was energetically stirred and heated on a water bath until it underwent a color transition from clear to wine-red, signifying the construction of β-CD-AuNPs. To prepare Au_core_@Ag_shell_ NPs, an amount of 0.4 mL of AgNO_3_ (10 mM) was inserted gradually into 50 mL of β-CD-AuNPs colloidal dispersion. After five minutes of incubation, the reaction mixture was heated on a water bath. After approximately half an hour, the wine-red color of the reaction mixture changed to a brownish yellow, signifying the creation of β-CD-Au_core_@Ag_shell_ NPs. Upon particle formation, the solution’s original wine-red color gradually faded while a yellow color developed. No extra NaOH or β-CD was mixed to trigger the chemical reduction of Ag^+^ ions and their deposition on the surface of β-CD-AuNPs. AuNPs were coated with Ag^+^ ions due to the alkalinity of the β-CD-AuNPs, which promoted their chemical reduction.

### 2.3. General Protocol for the Catalytic Reaction

A traditional cuvette cell was used to gather more information about catalytic reduction processes. In the cuvette cell, 2.70 mL of water and 0.5 mL of NB (5 mM) were mixed with 0.25 mL of freshly prepared NaBH_4_ (100 mM). Upon the addition of 0.05 mL of β-CD-Au_core_@Ag_shell_ NP colloidal catalysts to the solution mentioned above, we tracked the catalytic reduction process. The UV-vis absorbance spectral analysis was employed in the wavelength range of 200 to 500 nm at different time intervals.

### 2.4. General Protocol for Sensing Studies

Spectrophotometric and colorimetric detection of the Cyst was performed at 25 °C. Stock solution (0.01 mM) Cyst was prepared in PBS. Different quantities of Cyst were introduced by pipetting an aliquot of the stock solution into the conventional cuvette cell containing 2.5 mL of β-CD-Au_core_@Ag_shell_ NPs, and the subsequent mixture was shaken well. Following a 3-min incubation, absorption spectroscopy was used to evaluate the samples. A similar methodology was used for specificity studies, but potential interfering molecules were inserted for sensing specificity tests as alternatives to Cyst. Each set of experiments was performed a minimum of five times.

### 2.5. Protocol for Real Samples Investigation

The human urine samples were collected from Sigma Aldrich (St. Louis, MO, USA). In a 5 mL glass tube, 0.05 mL of urine samples were diluted with 10-fold PBS (10 mM, pH = 8) and then equilibrated for 30 min at room temperature for the actual sample analysis. Then, using the conventional addition approach, Cyst of two distinct known concentrations was added to the human urine samples. Then, the spiked specimens were introduced to the colloidal β-CD-Au_core_@Ag_shell_ NPs to analyze the absorption spectra. We used the calibration curve for a comparison required for concentration recovery evaluation to determine the concentrations of the specimen and the spiked ones. The ratio between the spiked amount and the retrieved one was used to determine the recovered amount of Cyst [46,47].

## 3. Results and Discussion

### 3.1. Characterization of β-CD-Au_core_@Ag_shell_ NPs

**Figure 1:**
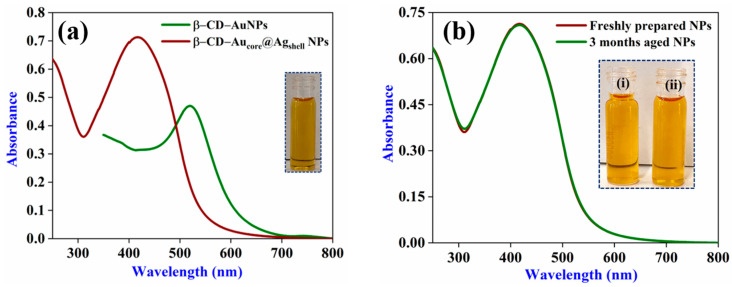
Absorbance spectra (**a**) of β-CD-Au_core_@Ag_shell_ NPs and β-CD-AuNPs while the inset shows the photo of freshly prepared β-CD-Au_core_@Ag_shell_ NPs. (**b**) absorbance spectra of freshly prepared and 3-month-aged β-CD-Au_core_@Ag_shell_ NPs while the insets of (i) and (ii) show the photos of freshly prepared and 3-month-aged β-CD-Au_core_@Ag_shell_ NPs.

Au_core_@Ag_shell_ NPs were primarily characterized and quantified using the UV-vis spectrophotometry for monitoring the LSPR spectral band to which we could attribut the observed variation in visual color. Since the dimensional shell-to-core ratio of Au_core_@Ag_shell_ NPs had a significant influence on the LSPR absorption band, even a small alteration in this metal ratio frequently results in a noticeable color and/or spectral change. The UV-vis absorbance spectra of colloidal β-CD-Au_core_@Ag_shell_ NPs and β-CD-AuNPs are given in Figure 1a. β-CD-AuNPs display a sharp single SPR band at 520 nm [45]. However, after the addition of AgNO_3_ into β-CD-AuNPs, the color of the β-CD-Au_core_@Ag_shell_ NPs changed to brownish yellow in color, while the LSPR band drastically blue-shifted from 520 nm to 406 nm (Figure 1a and Figure S1). This indicated that the Ag_shell_ was successfully deposited on the surface of Au_core_. The absorption peak occurred at 406 nm, corresponding to the formation of β-CD-Au_core_@Ag_shell_ NPs. Another crucial quality was the stability of β-CD-Au_core_@Ag_shell_ NPs. Analytical results that were falsely positive or falsely negative could have arisen from β-CD-Au_core_@Ag_shell_ NPs if they had not been stable enough. To further monitor the stability of the β-CD-Au_core_@Ag_shell_ NPs, we thus employed UV–visible spectroscopy for the changes in colorimetric features and LSPR band of Au_core_@Ag_shell_ NPs. Comparing the colloidal dispersion aged for six months to the freshly generated β-CD-Au_core_@Ag_shell_ NPs produced no considerable change in the LSPR peak at around 406 nm (Figure 1b). This demonstrated the exceptional stability of the β-CD-Au_core_@Ag_shell_ NPs in the state in which they were initially produced.

**Figure 2:**
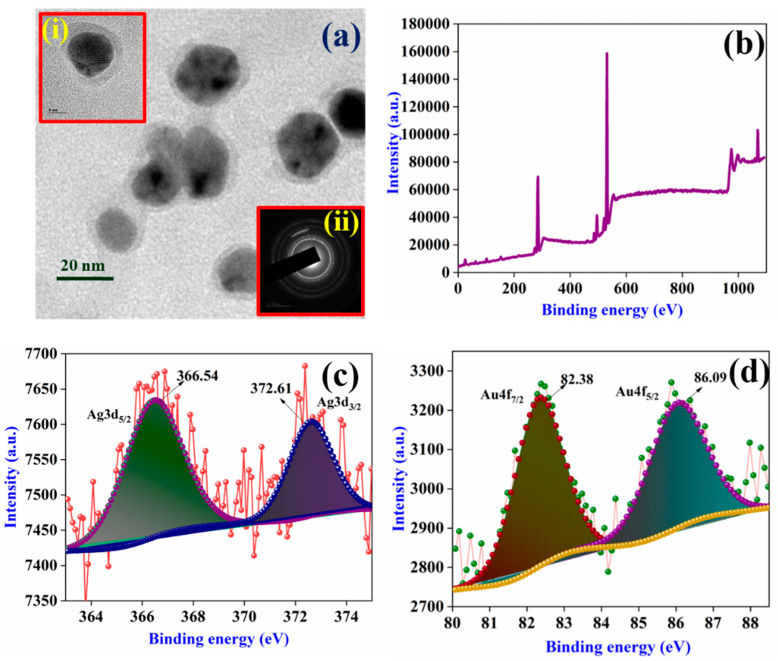
Characteristics of β-CD-Au_core_@Ag_shell_ (**a**) HR-TEM image. Inset of (**a**) shows a single β-CD-Au_core_@Ag_shell_ NP (i) and the corresponding SAED pattern (ii). (**b**) XPS survey spectrum, (**c**) deconvoluted spectrum of XPS for Ag, and (**d**) deconvoluted spectrum of XPS for Au.

HR-TEM micrographs were taken for the size and morphology of β-CD-Au_core_@Ag_shell_ NPs, as shown in Figure 2a. The electron microscope photographs revealed that the element of a lower atomic number (Ag) was seen in a bright gray color, whereas the element of a greater atomic number (Au) was seen in a darker gray color [48]. These two distinct brightnesses confirmed the presence of the core and shell bimetallic nanostructure, i.e., Au_core_@Ag_shell_ (the inset of Figure 2ai gives an example of the structure). β-CD-Au_core_@Ag_shell_ NPs were seen to have a roughly spherical shape with an average size of about 24 ± 4 nm. According to the results of the selective area energy diffraction (SAED) pattern, the Debye Scherrer rings indicated the crystallinity of β-CD-Au_core_@Ag_shell_ NPs (see the inset of Figure 2aii) [49].

The presence of β-CD molecules on the surface of Au_core_@Ag_shell_ NPs was verified by FT-IR spectral studies. The fact that β-CD had a high number of polar stabilizing groups, typically over 20 hydroxyls per β-CD molecule, meant it lent itself to being an excellent functionalization agent. FTIR results were provided for β-CD and β-CD-Au_core_@Ag_shell_ NPs for comparison, as seen in Figure S2. It was revealed that β-CD-Au_core_@Ag_shell_ NPs had two IR frequencies of around 2048 and 1328 cm^−1^. These frequencies were ascribed to the carboxylic acid groups that were generated in synthesis, forming the negatively charged surface of Au_core_@Ag_shell_ NPs [44,50,51]. The β-CD-Au_core_@Ag_shell_ NPs (Figure S2) were featured with the predominance of the β-CD vibrations, and the variation in peak location and intensities, supporting the existence of β-CD. The FTIR results indicated that gold nanoclusters had β-CD bound to their surface.

In addition, the surface composition of β-CD-Au_core_@Ag_shell_ NPs was characterized by XPS analysis. The XPS full scan survey spectrum illustrated the existence of Ag, Au, and C elements (around 285 eV), as shown in Figure 2b. It can also be seen from Figure 2c,d that the XPS spectra of Au_core_@Ag_shell_ NPs clearly showed strong peaks of Ag atoms, indicating that the outer shell consisted of Ag atoms [45,51]. The Au element, however, exhibited a very low intensity and a relatively small area under the peak because photoelectrons could only penetrate ~5 nm deep into the surface. The zero valence of the Ag atomic structure (Ag0) was demonstrated by the separation of the two spin-orbital peaks by 5.98 eV, which corresponded to the binding energies of Ag 3d5/2 and Ag 3d3/2, for the two peaks. The separation between these two spin-orbital peaks, i.e., 5.99 eV, demonstrated the zero valence of the Au atomic structure (Au0). The binding energies of Au 4f7/2 and Au 4f5/2 were found to be roughly 88.2 and 92 eV, respectively. The energies for Au and Ag agreed well with the binding energies found in the literature [52,53,54]. Instead of a hollow core forming inside the gold shell, these findings supported the establishment of an inverted core-shell structure.

**Sensing ability of Cyst by β-CD-Au_core_@Ag_shell_ NPs as a plasmonic sensor platform**.

### 3.2. Optimizing Sensing Parameters

Since the introduction of Cyst changed the absorbance at 602 and 404 nm, their ratio, i.e., A_602_/A_404_, also changed. In other words, higher concentrations of Cyst produced monotonically increased the ratio of A_602_/A_404_, which could be utilized for quantitative sensing of Cyst. It was found that the amount of Au_core_@Ag_shell_ NPs, solution pH, and Cyst interaction time were the important parameters for sensing optimization.

Under Cyst of 200 nM injected, the amount of β-CD-Au_core_@Ag_shell_ NPs was first examined for sensing optimization. As shown in Figure S3, the ratio maximized at 2.5 mL of β-CD-Au_core_@Ag_shell_ NPs. This led us to choose 2.5 mL of β-CD-Au_core_@Ag_shell_ NPs for the Cyst sensing. Second, the solution pH value was examined to maximize the ratio, A_602_/A_404_. However, the ratio varied to some extent in the pH range of 7.0–11.0, as seen in Figure S4. This led us to choose pH 8 where the ratio A_602_/A_404_ was maximized. It should be noted that the pH 8 coincided with the physiological pH level. Last, we examined the effect of the Cyst interaction time. The ratio A_602_/A_404_ was measured in time with a temporal step of 2 min, showing that the ratio saturated at the temporal phase as early as 2 min, beyond which no further increase in the ratio occurred (Figure S5). As a result, we decided to use a Cyst interaction time of 2 min.

### 3.3. Colorimetric Determination of Cyst Using β-CD-Au_core_@Ag_shell_ NPs as a Plasmonic Nanoprobe

**Figure 3:**
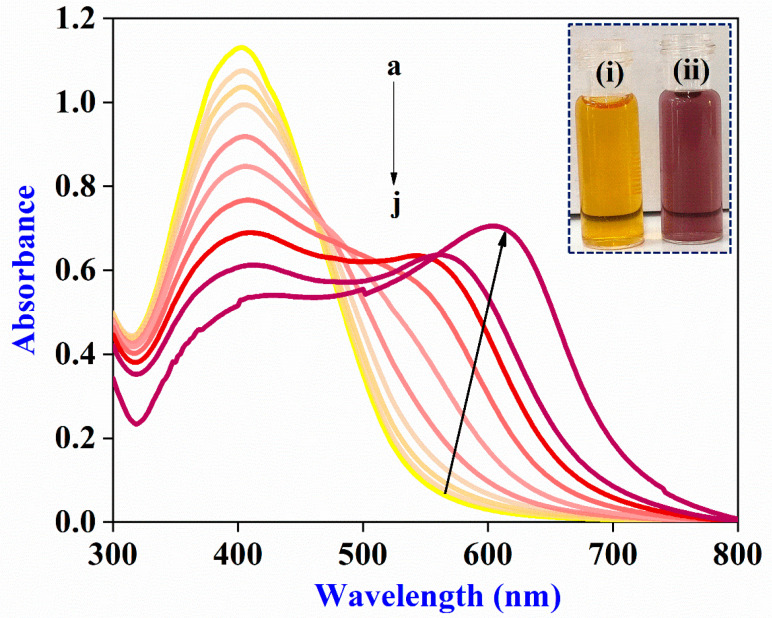
Absorbance changes for β-CD-Au_core_@Ag_shell_ NPs with various concentrations of Cyst in PBS (pH = 8.0). [Cyst] = (a) 0, (b) 25, (c) 50, (d) 75, (e) 100, (f) 200 (g) 300, (h) 400, (i) 500, and (j) 750 nM and the inset shows photographs of before (i) and after (ii) addition of (500 nM) Cyst into β-CD-Au_core_@Ag_shell_ NPs.

Figure 3 shows the change in the absorbance spectra of the β-CD-Au_core_@Ag_shell_ NPs colloid as various concentrations of Cyst were injected. A noticeable color shift occurred from brownish yellow to wine-red, as shown in Figure 3. The characteristic LSPR peak of colloidal β-CD-Au_core_@Ag_shell_ NPs was observed at 404 nm in the absence of Cyst. However, as higher concentrations of Cyst solution were introduced, the LSPR band intensity decreased at about 404 nm, while a new peak band appeared at about 600 nm. Such a huge change in LSPR peak (about 200 nm) was usually not due to the refractive index change of local media around NPs but was probably due to the aggregation of β-CD-Au_core_@Ag_shell_ NPs (more details are given in the next section).

### 3.4. Cyst Sensing Mechanism by β-CD-Au_core_@Ag_shell_ NPs

As mentioned above, the NPs aggregation suspected to result in the color shift of the LSPR peak could be elucidated in two ways. First, upon the introduction of thiol-containing Cyst, the weakly adsorbed β-CD molecules on the surface of Au_core_@Ag_shell_ NPs were easily replaced by a metal–thiol bond formation. This could subsequently cause the Au_core_@Ag_shell_ NPs to aggregate. Second, β-CD-Au_core_@Ag_shell_ NPs were inherently negatively charged due to the secondary hydroxyl groups. This maximized the electrostatic repulsion between them, preventing aggregation. However, as the positively charged Cyst molecules interacted electrostatically with them, the diminished electrostatic repulsion between them rendered them less aggregated than before the Cyst injection. Cyst detection by similar NPs aggregation could also be found in [13,42,43].

To verify the occurrence of the NP aggregation, we measured the Fourier transform infrared (FTIR) spectra of two cases, i.e., a case of Cyst only and another case of Cyst-induced β-CD-Au_core_@Ag_shell_ NPs aggregates. As shown in Figure S6, the infrared (IR) peak was shown at the stretching frequency of the thiol group, i.e., 2500 cm^−1^, clearly manifesting pristine Cyst. Meanwhile, the broad peak was seen at the IR stretching frequency of amino functional groups in the range of 3000 to 3500 cm^−1^ [14,55]. Surprisingly, the case of Cyst plus β-CD-Au_core_@Ag_shell_ NP showed no peak of the thiol stretching vibration near 2500–2600 cm^−1^ [13,14,42], reflecting a strong metal–thiol bonding-induced aggregation of NPs. This illustrated the Cyst sensing mechanism based on LSPR peak change due to NP aggregation mediated by metal–thiol bonding.

**Figure 4:**
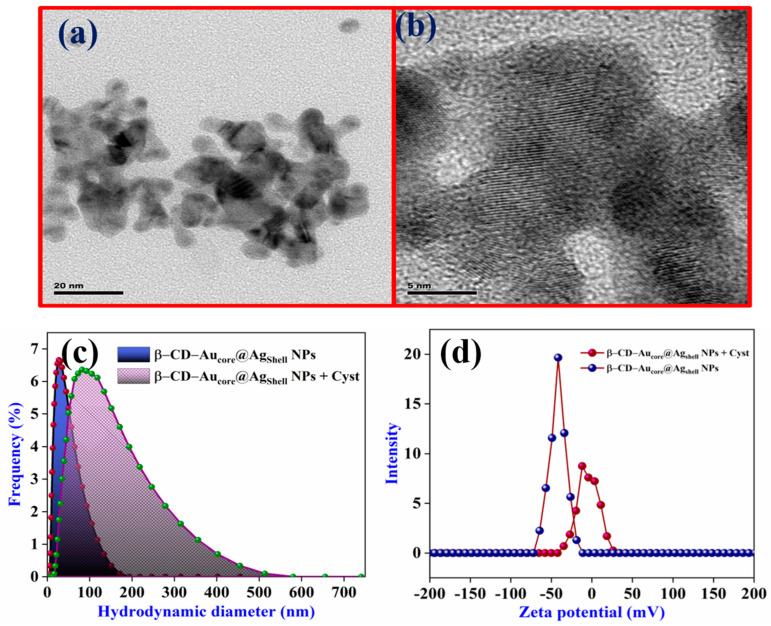
HR-TEM images of β-CD-Au_core_@Ag_shell_ NPs that interacted with Cysts at different magnifications (**a**,**b**). Comparison between β-CD-Au_core_@Ag_shell_ NPs that interacted with and without Cyst, by measurement of DLS (**c**) and Zeta potential (**d**).

Cyst-induced NP aggregation was further verified by high-resolution transmission electron microscopy (HR-TEM), dynamic light scattering (DLS), and zeta potential measurement. Figure 4a shows the HR-TEM picture of the β-CD-Au_core_@Ag_shell_ NPs that underwent interaction with Cyst molecules. It exhibited the aggregated structure with an average size increase from 24 ± 4 nm to 45 ± 5.8 nm. Furthermore, the aggregated NPs in a solution medium were checked by DLS, as shown in Figure 4b. Prior to interaction with Cyst, β-CD-Au_core_@Ag_shell_ NPs were measured to have an average hydrodynamic diameter of ~30 nm. However, their interaction with Cyst enlarged the averaged diameter from ~30 nm to 108.3 nm, as seen in Figure 4c.

Additionally, we measured the zeta potential of β-CD-Au_core_@Ag_shell_ NPs alone and those interacted with Cyst molecules, as shown in Figure 4d. β-CD-Au_core_@Ag_shell_ NPs displayed a zeta potential value of −42.40 mV while β-CD-Au_core_@Ag_shell_ NPs that interacted with Cyst molecules produced a zeta potential of −10.12 mV. This significant drop in the NP negative surface charge supported the diminished repulsion between NPs, which is likely to result in more chance of NP aggregation. This was due to the significant electrostatic attraction between the positively charged Cyst and the negatively charged β-CD-Au_core_@Ag_shell_ NPs, as the positively charged Cyst was found to have an isoelectric point of 9.24 [13,43,56]. Altogether, all the results from HR-TEM, DLS, and zeta potential measurements were in agreement for NP aggregation as a result of β-CD-Au_core_@Ag_shell_ NPs interaction with Cyst.

### 3.5. Analytical Performance

**Figure 5:**
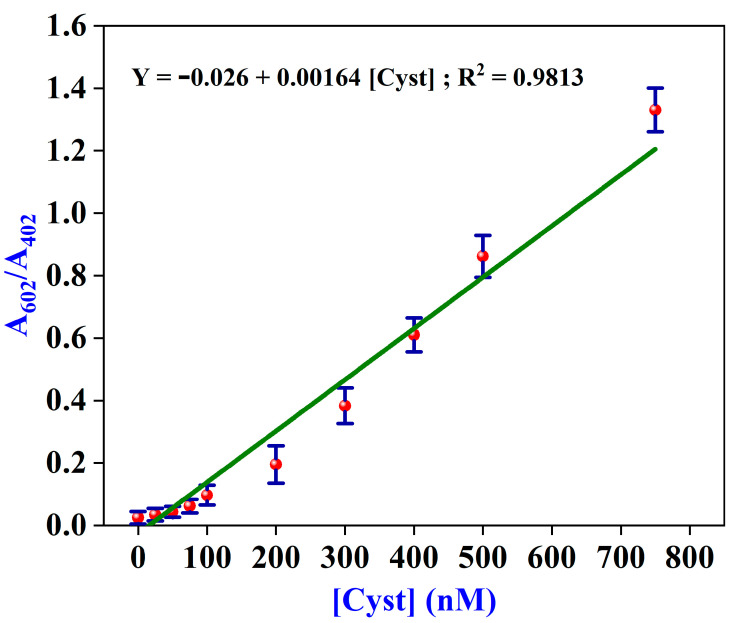
Calibration plot between the A_602_/A_404_ and different concentrations of Cyst.

As mentioned above, thiol-containing Cyst added to colloidal β-CD-Au_core_@Ag_shell_ NPs caused them to aggregate, consequently altering the LSPR band and thus the absorbance ratio A_602_/A_404_. Figure 5 shows the rise of the ratio as a function of Cyst introduced with a concentration range of 25 to 750 nM. The linear regression that fitted to the data was obtained with R^2^ of 0.9813, enabling the calibration to be available for quantitative sensing of Cyst with an LOD of 1.83 nM.

Table S1 provides a summary of the sensing characteristics between various analytical methods reported in the literature for comparison with this study, such as reaction time and LOD. The reaction time of 2 min taken for sensing Cyst with our nanoprobes proved to be markedly rapid without compromising sensitivity or straightforwardness of the protocol of nanoprobe production [13,41,42,43].

### 3.6. Sensing Specificity

**Figure 6:**
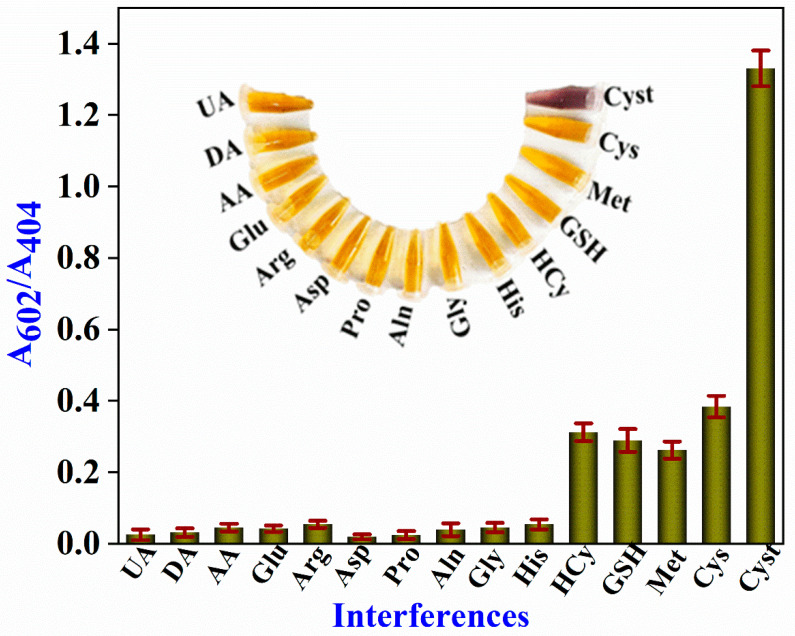
Sensing specificity of the β-CD-Au_core_@Ag_shell_ NPs to Cyst molecules against other interfering molecules such as uric acid (UA), dopamine (AA), ascorbic acid (AA), glutamate (Glu), arginine (Arg), asparagine (Asp), proline (Pro), alanine (Ala), glycine (Gly), histidine (His), Hcy, GSH, Met, and Cys in PBS (pH = 8.0).

Competitive assays were conducted to investigate the sensing specificity of β-CD-Au_core_@Ag_shell_ NPs toward Cyst detection against other possible interfering molecules. The degree of NP aggregation that arose with the ratio of A_602_/A_404_ was estimated when using various interfering molecules for the specificity test, as shown in Figure 6. It was noted that Cyst concentrations less than 750 nM were sufficient to cause aggregation of β-CD-Au_core_@Ag_shell_ NPs, leading to a notably higher absorption ratio. However, when introducing other types of thiol-containing amino acids with a concentration of 15 μM, such as cysteine (Cys), homocysteine (HCy), methionine (Met), and glutathione (GSH), the absorption ratio changed in no more than about 20% of Cyst cases (750 nM) as seen in Figure 6. Meanwhile, when using the other types of interfering molecules, a negligible rise in the absorption ratio was observed by increasing their concentration to 75 μM, which was about 100 times larger than the Cyst concentration. The results proved that the β-CD-Au_core_@Ag_shell_ NPs-based colorimetric sensors were exceedingly specific to Cyst, encouraging their use as nanoprobes for on-site real-time visual checks of the presence of Cyst, as shown by the exclusive color change in the Cyst case only, which is shown in the inset of Figure 6.

### 3.7. Investigation for Real-World Applications

To demonstrate the applicability of the abovementioned techniques for the quantitative sensing of Cyst present in human urine, we diluted human urine purchased from Sigma Aldrich, St. Louis, Missouri, United States of America, PBS (pH = 8.0) by 10-fold, spiked Cyst to make up three known concentrations (100, 300, and 500 nM) of Cyst, and applied the aforementioned colorimetric techniques with β-CD-Au_core_@Ag_shell_ NPs for sensing them quantitatively. The results are listed in Table S2. When the spiked Cyst concentration fell within or near the linear range over which the linear regression-based calibration was made (Figure 6), more than 97% recovery was obtained, manifesting great sensing accuracy. Although the spiked concentration was made well beyond the upper limit of the linear regression, i.e., 500 nM, recovery was maintained at over 95%. This indicated that the plasmon-based colorimetric technique for Cyst sensing with nanoprobes, i.e., β-CD-Au_core_@Ag_shell_ NPs, may be very promising for the application of sensing Cyst moieties in a human-derived liquid such as human urine, in a reliable, accurate, and very straightforward manner.

### 3.8. Catalytic Conversion of NB to Aniline

**Figure 7:**
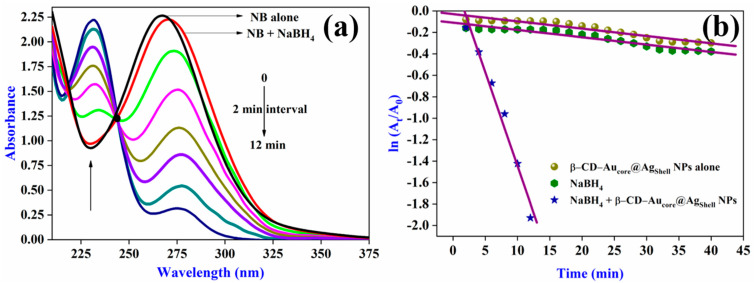
Time-evolution of absorbance spectra (**a**) representing the catalytic conversion of nitrobenzene to aniline using NaBH_4_ with the nanocatalysts of β-CD-Au_core_@Ag_shell_ NPs and the plot of the corresponding kinetics (**b**).

We examined the catalytic activity of β-CD-Au_core_@Ag_shell_ NPs for the chemical conversion of NB to aniline in the presence of NaBH_4_ in an aqueous media. The catalytic hydrogenation progress was closely monitored in time with a progressive step of 2 min, using UV-Vis absorbance spectroscopy. Figure 7 shows the continual measurement of absorbance spectra as chemical reactions evolved for a solution composed of β-CD-Au_core_@Ag_shell_ NPs, NaBH_4_, and NB in water. Before the addition of NaBH_4_ and β-CD-Au_core_@Ag_shell_ NPs, NB displayed an absorption band of around 268 nm, as seen in Figure 7a. NaBH_4_ by itself was known to be ineffective for reducing NB without a catalyst (Figure 7b), requiring a catalyst for the effective reduction of NB [57,58]. Since a predetermined quantity of β-CD-Au_core_@Ag_shell_ NPs was introduced to the reaction mixture, the absorption band at 272 nm gradually decreased in time while a new absorption band was concurrently created at 231 nm due to the conversion of NB to aniline (NB and aniline were represented by the absorption peaks seen at 272 and 231 nm, respectively) [50]. Additionally, the isosbestic point occurred at 244 nm, as seen in Figure 7), manifesting the equilibrium of the reactants and the products. These findings revealed that by promoting the catalytic hydrogenation of NB to aniline, the β-CD-Au_core_@Ag_shell_ NPs served as effective nanocatalysts. Within 12 min, around 87% of the NB molecule had converted to aniline. The subsequent alteration in the colors of the colloidal (NB + NaBH_4_) solution from slightly yellow to colorless offered a second phase during which NB was being catalytically reduced to aniline, verifying the catalytic conversion of NB into aniline. NaBH_4_ served as the hydride ion source in this catalytic reduction and could charge the β-CD-Au_core_@Ag_shell_ NP surfaces. Adsorption of both NaBH_4_ and NB on the β-CD-Au_core_@Ag_shell_ NP’s surface charged Au_core_@Ag_shell_ NPs and subsequently caused NB to be hydrogenated. NB molecules were reduced to the nitroso compound and it eventually became the equivalent hydroxylamine molecule rather quickly. Finally, aniline was generated by catalytically reducing the hydroxylamine molecule [44,57,58].

Monitoring the reduction of the NB absorbance peak allowed its reduction reaction to be easily tracked. No induction time was observed, revealing that the reactants bound to the active sites rapidly under quickly and uniformly dispersed β-CD-Au_core_@Ag_shell_ NPs. The reduction reaction follows pseudo-first-order kinetics because the concentration of NaBH_4_ was substantially larger than the concentration of NB. The rate constant was determined with the equation $-kt={log}_{e}(A_{t}/A_{o})$, whereas *k* denoted the rate constant, and *t* represented the time. $A_{o}$ and $A_{t}$ were the absorbance at initial time (t=0) and at reaction time t, respectively [36,55,56,57,58,59,60]. Fitting to the plots of $({log}_{e}A_{t}/A_{o})\;vs.\;t$ allowed the reaction rate to be determined with 0.18 min^−1^, as shown in Figure 7b, showing a very high reaction rate.

## 4. Conclusions

We fabricated, in quite a simple manner with a water solvent, the versatile nano-agents, i.e., β-CD-Au_core_@Ag_shell_ NPs, for the colorimetric quantitative sensors for Cyst present in human urine specimens, and for the catalytic conversion of NB to aniline. Cyst sensing could be eye-checked as the colloidal color changed from brownish yellow to colorless when increasing the Cyst concentration in the specimens. This was due to the change in the LSPR peak from a wavelength of around 400 nm to one of 600 nm, which caused the absorbance ratio A_602_/A_404_ to change. Such changes were ascribed to the NP aggregation that resulted from the metal–thiol bonding via the effective replacement of β-CD by Cyst. The remarkably rapid detection time, which was as short as 2 min, was demonstrated together with excellent specificity against other interfering molecules. While the detection linear range was 25–750 nM, the LOD was achieved as 1.83 nM, providing very good sensitivity. These nanosensors also demonstrated excellent accuracy in Cyst sensing.

The versatile feature of β-CD-Au_core_@Ag_shell_ NPs could also include another aspect, i.e., catalytic effects for the effective conversion reaction of NB to aniline by NaBH_4_. This technique with β-CD-Au_core_@Ag_shell_ NPs as nanocatalysts could also find use in the detoxication of aqueous media while producing useful chemicals at the same time. The abovementioned various features of β-CD-Au_core_@Ag_shell_ NPs demonstrated could find potential applications in cost-effective rapid screening in Cyst-relevant drug industries or sensors based on human urines, and in catalytic reactions for the detoxication of water system.

## Data Availability

All research data will be made available upon request.

## Supplementary Materials

The following supporting information can be downloaded at: https://www.mdpi.com/article/10.3390/bios14110544/s1. Scheme S1. Chemical structure of Cysteamine; Scheme S2. Schematic illustration of catalytic conversion of NB to aniline; Figure S1. The absorbance spectrum of freshly prepared β-CD-Au_core_@Ag_shell_ NPs. Inset shows the photo of their colloid; Figure S2. FT-IR spectra of β-CD alone and β-CD-Au_core_@Ag_shell_ NPs; Figure S3. Optimization of absorbance ratio, i.e., A_602_/A_404_ by adjusting the amount of β-CD-Au_core_@Ag_shell_ NPs under Cyst of 200 nM introduced (pH = 8, PBS); Figure S4. Effect of pH on the absorbance ratio A_602_/A_404_ for β-CD-Au_core_@Ag_shell_ NPs under Cyst of 200 nM introduced; Figure S5. The absorbance ratio between 602 nm and 404 nm as a function of interaction time (with Cyst concentration of 300 nM (pH = 8, PBS)); Figure S6. FT-IR spectra of Cyst alone and cyst-induced β-CD-Au_core_@Ag_shell_ NPs aggregates; Table S1. Comparison of present work with previously reported Cyst detection approaches [13,31,40,42,43,61,62,63]; Table S2. Quantification of Cyst in human urine samples by β-CD-Au_core_@Ag_shell_ NPs.
