# Supplementary material for: β-Cyclodextrin Functionalized Au@Ag Core-Shell Nanoparticles: Plasmonic Sensors for Cysteamine and Efficient Nanocatalysts for Nitrobenzene-to-Aniline Conversion
Source: Biosensors (Basel). 2024 Nov 9;14(11):544. doi: 10.3390/bios14110544 (PMC11591858; doi:10.3390/bios14110544)
Supplement: Supplementary file 1 [file biosensors-14-00544-s001.zip › biosensors-3261403-supplementary.pdf]

biosensors

Supporting Information

β-Cyclodextrin Functionalized Au@Ag Core-Shell Nanoparticles: Plasmonic Sensors for Cysteamine and Efficient Nanocatalysts for Nitrobenzene-To-Aniline Conversion

Ramar Rajamanikandan [1,2], Kandasamy Sasikumar [1,2] and Heongkyu Ju [1,2,*]

[1] Department of Physics, Gachon University, Seongnam-si 13120, Republic of Korea
[2] Gachon Bionano Research Institute, Gachon University, Seongnam-si 13120, Republic of Korea
* Correspondence: batu@gachon.ac.kr

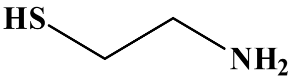

Scheme S1. Chemical structure of Cysteamine.

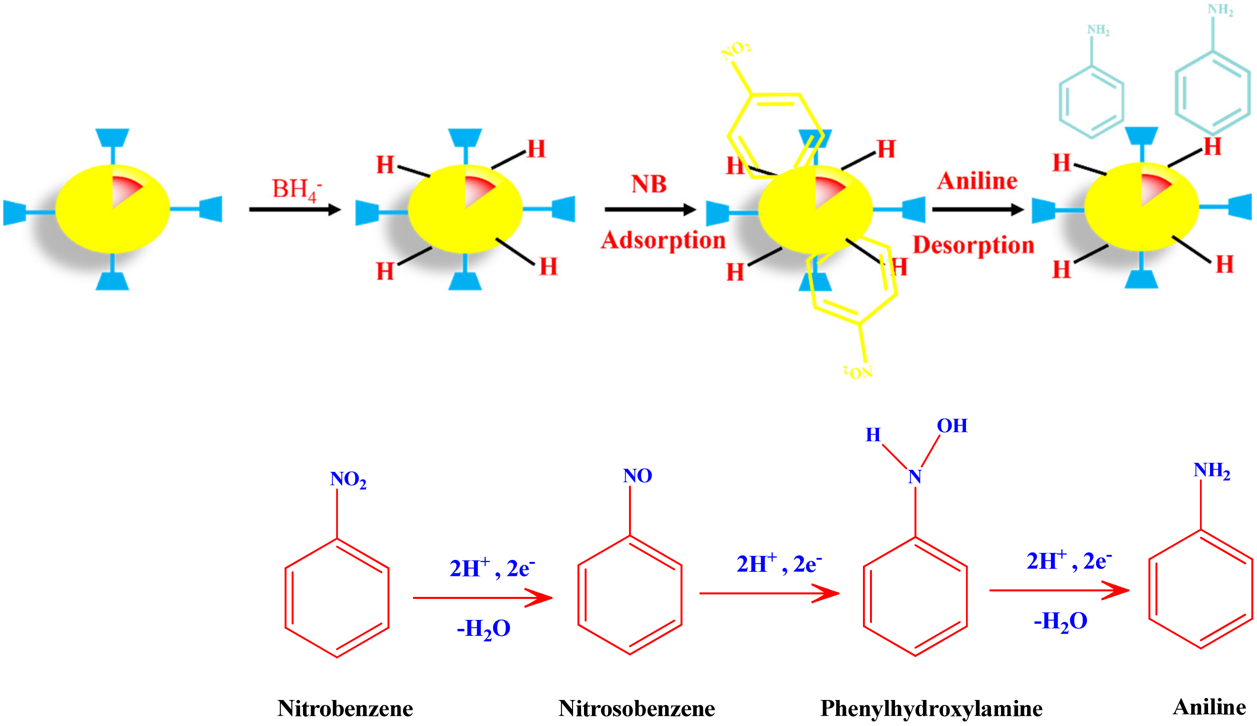

Scheme S2. Schematic illustration of catalytic conversion of NB to aniline.

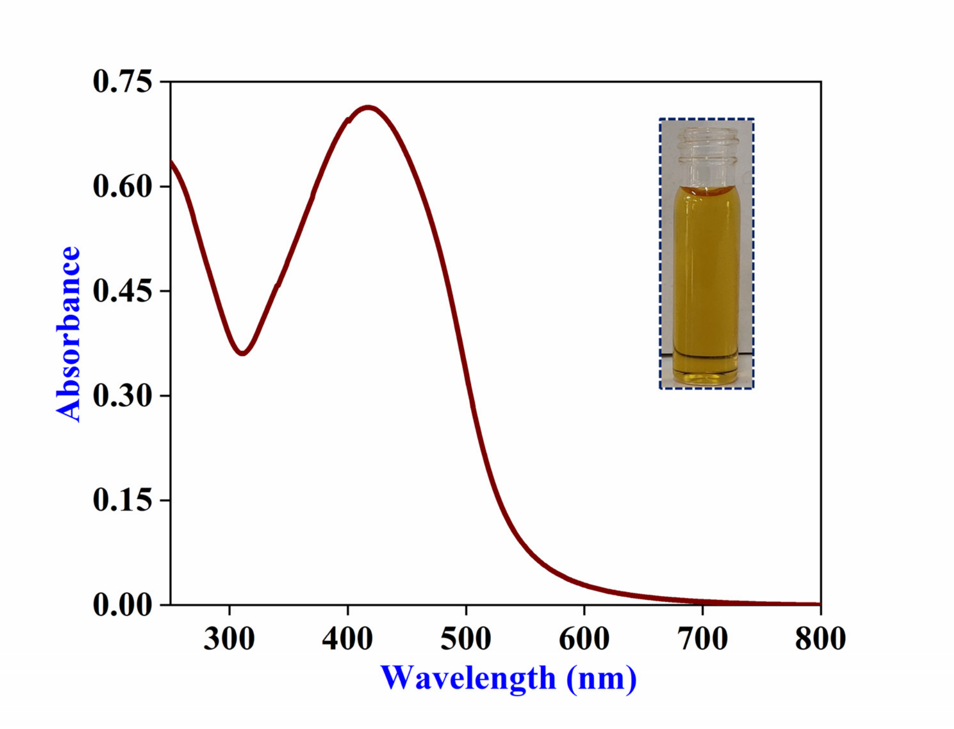

Figure S1. The absorbance spectrum of freshly prepared β–CD–Au_{core}@Ag_{shell} NPs. Inset shows the photo of their colloid.

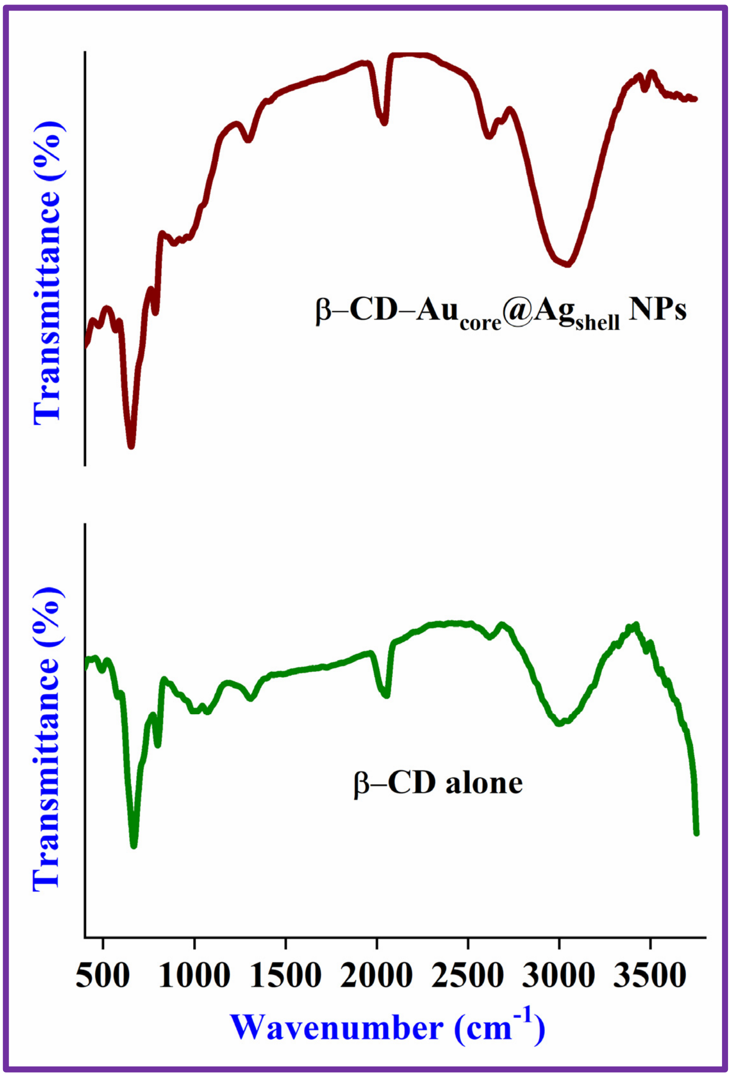

Figure S2. FT-IR spectra of β–CD alone and β–CD–Au$_{core}$@Ag$_{shell}$ NPs.

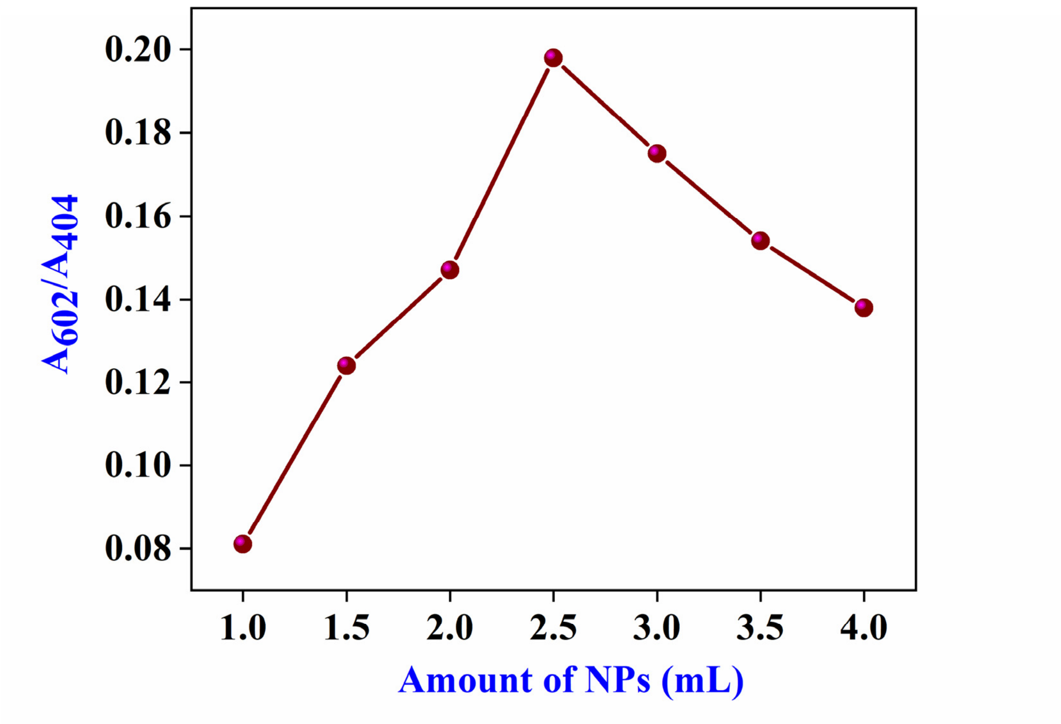

Figure S3. Optimization of absorbance ratio, i.e., A_{602}/A_{404} by adjusting the amount of β–CD–Au_{core}@Ag_{shell} NPs under Cyst of 200 nM introduced (pH = 8, PBS).

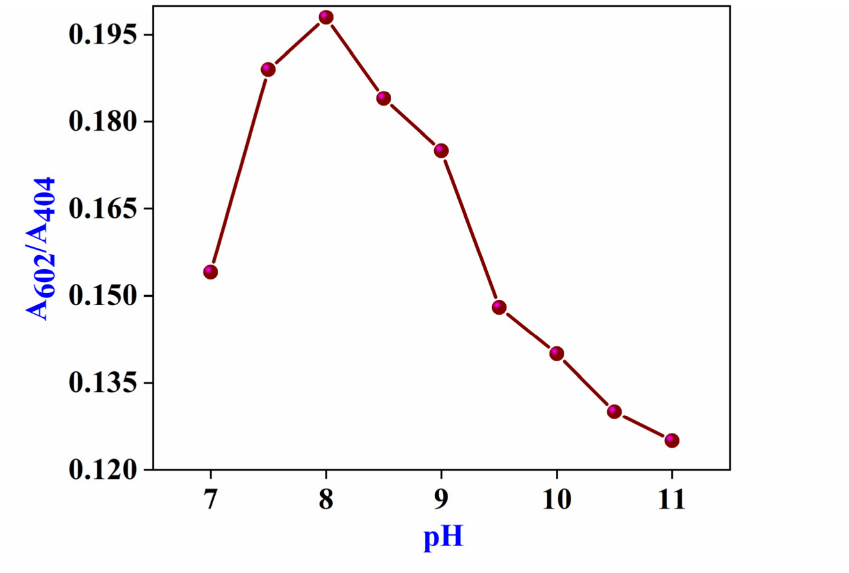

Figure S4. Effect of pH on the absorbance ratio A_{602}/A_{404} for β–CD–Au_{core}@Ag_{shell} NPs under Cyst of 200 nM introduced.

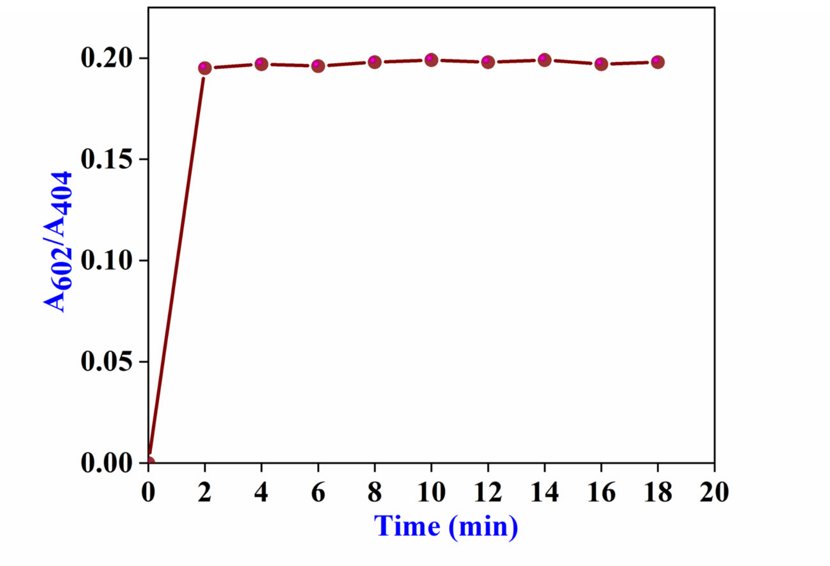

Figure S5. The absorbance ratio between 602 nm and 404 nm as a function of interaction time (with Cyst concentration of 300 nM (pH=8, PBS)).

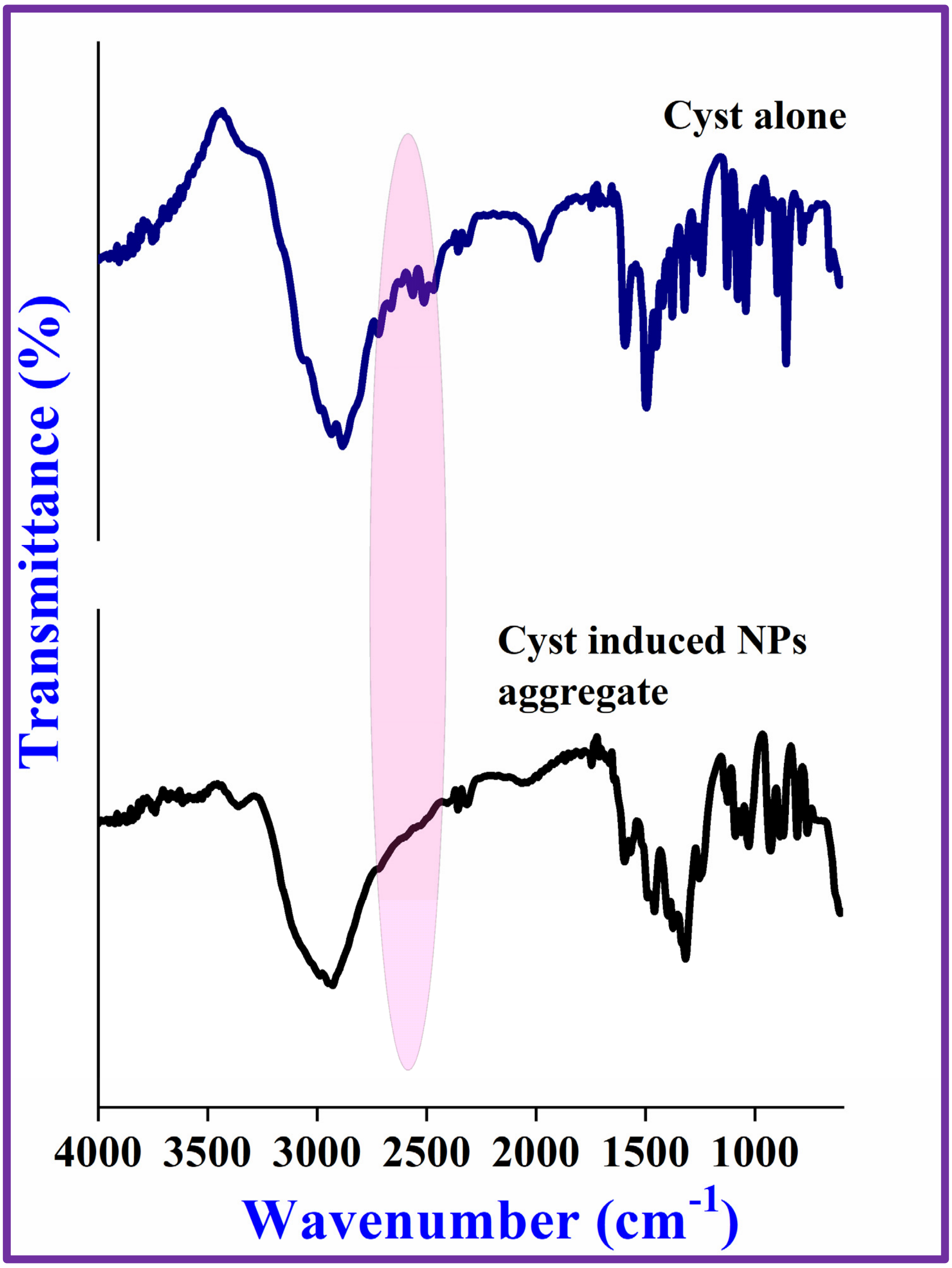

Figure S6. FT-IR spectra of Cyst alone and cyst-induced β–CD–Au_{core}@Ag_{shell} NPs aggregates.

Table S1. Comparison of present work with previously reported Cyst detection approaches.

Method	Material	Reaction time (min)	Linear range	LOD (nM)	Ref
Fluorescence	Bovine serum albumin stabilized gold nanoclusters	not mentioned	0.5-10 μM	150 nM	61
Fluorescence	Nitrogen-doped carbon dots	5	10-210 μM	75.6 nM	62
Fluorescence	D-penicillamine stabilized cadmium sulfide quantum dots	15	2-10 μM	70 nM	63
Fluorescence	Carbon dots stabilized AgNPs	30	2-16 μM	350 nM	31
Colorimetry	Polyvinyl pyrrolidine-AgNPs	15	0.1-1 μM	4.9 nM	13
Colorimetry	AuNPs	2	0.01-0.05 μM	100 nM	40
Colorimetry	Citrate-Au@Ag core-shell NPs	10	0.1-0.8 μM	0.33 nM	42
Colorimetry	AgNPs	5	0.6-1.8 μM	0.37 μM	43
Colorimetry	β−CD−Au_{core}@Ag_{shell} NPs	2	25-750 nM	1.83 nM	This work

Table S2. Quantification of Cyst in human urine samples by β−CD−Au_{core}@Ag_{shell} NPs.

Sample Type	Cyst spiked (nM)	Cyst found (nM)	Recovery (%)	RSD
Human urine specimens	100	98.4	98.4	2.35
	300	292.5	97.5	1.98
	500	479.0	95.8	3.54

RSD; Relative standard deviation
